# Supplementary material for: Nature of the Insulating Ground State of the Two-Dimensional Sn Atom Lattice on SiC(0001)
Source: Sci Rep. 2016 Jul 28;6:30598. doi: 10.1038/srep30598 (PMC4964574; doi:10.1038/srep30598)
Supplement: Supplementary Information [file srep30598-s1.pdf]

Nature of the Insulating Ground State of the Two-Dimensional Sn Atom Lattice on SiC(0001)

Seho Yi[1], Hunpyo Lee[2], Jin-Ho Choi[1,3] & Jun-Hyung Cho[1,*]
[1]*Department of Physics and Research Institute for National Sciences, Hanyang University 17 Haengdang-Dong, Seongdong-Ku, Seoul 133-791, Korea*
[2]*School of General Studies, Kangwon National University, 346 Jungang-ro, Samcheok-si, Kangwon-do, Korea*
[3]*Research Institute of Mechanical Technology, Pusan National University, 30 Jangjeon-dong, Geumjeong-gu, Pusan 609-735, Korea*

1. Atomic geometry and band structure of the NM 3×3 structure

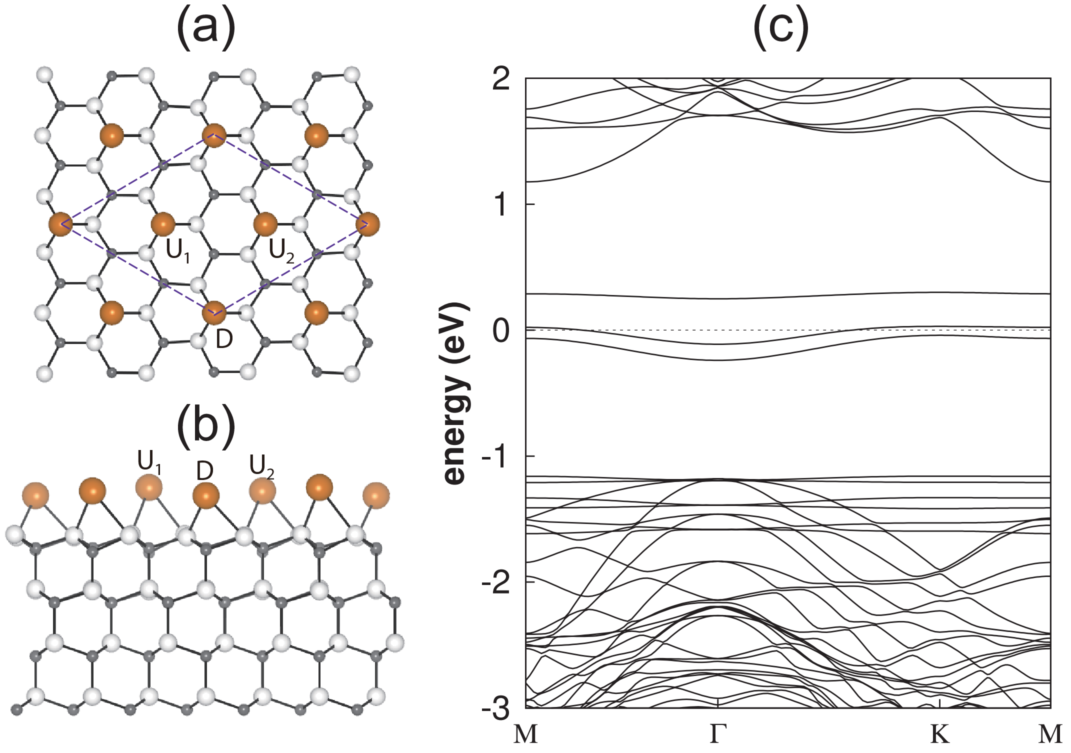

Figure S1: (a) Top and (b) side views and (c) the band structure of the NM 3×3 structure obtained using the LDA calculation. The dashed line indicates the 3×3 unit cell, where three Sn atoms of different heights are designated as U_1, U_2, and D. Here the position of U_1 (U_2) is higher than that of D by 0.38 (0.22) Å. The energy zero in (c) represents the Fermi level. The band structure is seen that there is a partially-filled band crossing the Fermi level, indicating a metallic feature.
